# Plant growth-promoting properties of the phosphate-solubilizing red yeast *Rhodosporidium paludigenum*

**DOI:** 10.1007/s11274-022-03498-9

**Published:** 2022-12-24

**Authors:** Yi-Ru Chen, Chih-Yen Kuo, Shih-Feng Fu, Jui-Yu Chou

**Affiliations:** Department of Biology, National Changhua University of Education, Changhua City, 500 Taiwan

**Keywords:** Biofertilizer, Calcium phosphate, Phosphate-solubilizing microorganisms (PSMs), Red yeast, *Rhodosporidium paludigenum*

## Abstract

Phosphorus (P) is one of the essential elements that are necessary for plant development and growth. However, the availability of soluble forms of P for plants in the soils is limited, because a large proportion of it is bound to soil constituents. Thus, the concentration of P available to plants at any time is very low and, moreover, its availability depends on the soil pH. As a solution, phosphate-solubilizing microorganisms (PSMs) are employed that render inorganic P available to plants in soluble form. Thus far, research into PSMs has been insufficient, and only few such organisms have been considered for exploitation as microbial fertilizer strains. The characteristics of plant growth promotion with the plant-PSMs coculture system remain to be elucidated. In the current study, we report on the isolate *Rhodosporidium paludigenum* JYC100 that exhibits good performance for solubilizing calcium phosphate. We found that it can be regulated by the amount of soluble phosphate. Furthermore, *R*. *paludigenum* JYC100 promotes plant growth under specific conditions (P deficiency, but with insoluble phosphate) in different media and soil pots. In contrast, the yeast *Aureobasidium pullulans* JYC104 exhibited weak phosphate-solubilizing capacities and no plant growth-promoting ability. Compared to control plants, the biomass, shoot height, and cellular inorganic P content of plants increased in plants cocultivated with *R*. *paludigenum* JYC100. In addition, histochemical GUS and qRT-PCR assays of phosphate starvation-induced (PSI) genes showed that the transcript levels of these PSI genes are decreased in the plants cocultured with *R*. *paludigenum* JYC100. These findings reflect the unique ability of *R*. *paludigenum* JYC100 to convert insoluble P compounds to plant-available P, thereby leading to growth promotion. Our study results highlight the use of yeasts as potential substitutes for inorganic phosphate fertilizers to meet the P demands of plants, which may eventually improve yields in sustainable agricultures.

## Introduction

Pi (inorganic phosphate) deficiency is a major nutritional problem faced by plants in many agro-ecosystems, and plants respond to Pi deficiency via the starvation signaling pathway that regulates the expression of Pi-starvation-induced (PSI) genes (Puga et al. 2017). In higher plants, the family of phosphate transporters (PHTs) mediates the uptake and translocation of Pi (Raghothama 1999). Phosphate transporters can be divided into phylogenetically distinct subfamilies, designated PHT1–PHT4. The plant PHT1 subfamily belongs to the Pi/H + symporter system that mediates Pi acquisition across the plasma membrane of root cells (Zhang et al. 2014). The *Arabidopsis* AtPHT1 gene family contains nine members (*AtPHT1;1*–*AtPHT1;9*), among which *AtPHT1;1* and *AtPHT1;4*, also named *PT1* and *PT2*, are high-affinity Pi transporters (PTs) and play major roles in Pi acquisition from the soil to the roots in both low- and high-P environments (Misson et al. 2004; Shin et al. 2004). *AtPHT1;8* and *AtPHT1;9* are likely to mediate Pi acquisition by roots only during P starvation (Remy et al. 2012).

In response to persistent phosphorus (P) deficiency, plants have evolved adaptive mechanisms to acquire and recycle Pi to cope with Pi stress conditions. In addition to facilitating the acquisition of Pi by PHTs, Pi deficiency also triggers the re-modelling of phospholipids, which serve as an important pool of organic Pi for plants (Wang and Tontonoz 2019). In *Arabidopsis*, a member of the phospholipase D gene family (*PLDZ2*) is induced upon Pi starvation in both shoots and roots. Phosphatidylcholine (PtdCho) and phosphatidylethanolamine (PtdEA) are the most abundant membrane phospholipids. To recycle the endogenous P from P-containing molecules, *PLDZ2* participates in the hydrolysis of PtdCho and PtdEtn to release Pi and to provide diacylglycerol (DAG) for the synthesis of galactolipids (Cruz-Ramirez et al. 2006).

*Arabidopsis* inositol pentakisphosphate 2-kinase, *AtIPK1*, modulates phosphate homeostasis via transcriptional regulation for growth (Kuo et al. 2014). The enzyme catalyzes the last step in the biosynthetic pathway of phytic acid, called phytate, which has been detected in a wide range of plant tissues as a P reservoir (Sun et al. 2007). Besides, the glycerol-3-phosphate permease (G3Pp) family, comprising five members (AtG3Pp1 to 5) in *Arabidopsis*, is another group of Pi starvation-responsive genes (Ramaiah et al. 2011). Among the member of this gene family, glycerol-3-phosphate permease 1 (*G3PP1*) has shown a 24-fold induction in the roots of Pi-deprived seedlings. However, the molecular mechanism underlying this process is not completely clear.

P plays a very important function in plants, as it is a component of the complex nucleic acid structure and regulates protein synthesis. It helps a plant convert other nutrients into usable building blocks that allow it to grow. Pi deficiency in plants generally leads to leaves and stems turning dark green and to a stunted appearance (Hammond et al. 2004). Fertilizer application is one of the methods for improving the availability of soil nutrients to plants. However, overuse of P fertilizer can cause dangerous levels of pollution in waterways that harm aquatic plants and animals. Phosphate-solubilizing microorganisms (PSMs) are therefore seen as an eco-friendly means of managing P deficiency to increase agricultural productivity (Tian et al. 2021). The principal mechanisms underlying Pi solubilization by PSMs include the secretion of organic acids, the production of enzymes, and the excretion of siderophores that can chelate metal ions and form complexes (Alori et al. 2017). These processes make largely immobile P reserves in soils available for uptake by plants. The phosphate-solubilizing potential of the rhizosphere microbial community has been shown to help plants acquire phosphates from soil (Mitra et al. 2020). Therefore, it is of great significance to study the effect of PSMs on the growth of plants for their future application in agriculture.

In this study, a P-solubilizing yeast, *Rhodosporidium paludigenum*, was isolated from a carnivorous plant, *Drosera spatulate*, in our previous study aimed to taking *Arabidopsis thaliana* and cherry tomato (*Solanum lycopersicum* var. *cerasiforme*) as the material and investigating the plant growth promoting effects by inoculation with this red yeast (Fu et al. 2016). The results of this study will provide basic data and practical guidance for the development and applications of phosphate-solubilizing *R*. *paludigenum* as a biological P fertilizer.

## Materials and methods

### Yeast culture condition

The yeast strains, *R*. *paludigenum* JYC100 and *Aureobasidium pullulans* JYC 104, used in this study were isolated from *Drosera spatulata* leaves in our previous study (Fu et al. 2016).

### In vitro screening of yeast for calcium-phosphate-solubilizing activity

For the phosphate solubilization assay, the selected yeast strains were screened in a Pi-deficient (−P) quarter-strength Murashige–Skoog (MS) medium (pH 5.7) containing 10 g sucrose, 0.5 g 2-morpholinoethanesulfonic acid monohydrate, 25 mL 10 × macronutrient salts (8.25 g NH_4_NO_3_, 9.5 g KNO_3_, 2.2 g CaCl_2_·2H_2_O, 1.85 g MgSO_4_·7H_2_O, and 500 mL deionized water), 25 mL 10 × minor nutrient salts (M0529, Sigma), 950 mL deionized water, solid medium with 1.2% (w/v) Bacto agar, and 5 g CaHPO_4_·2H_2_O (calcium phosphate dibasic dihydrate, CPDD Sigma) or Ca_5_(OH)(PO_4_)_3_ (calcium phosphate tribasic, CPT, Bio Basic Inc.). Yeast suspensions (3 µL) were pipetted in the center of (−P) MS agar plates. After 5 (MS-calcium phosphate dibasic dihydrate; MS-CPDD) or 7 days (MS-calcium phosphate tribasic; MS-CPT) of incubation at 28 °C, a visible halo zone around the colonies was observed (caused by the solubilization of calcium phosphate by the yeast). The solubilization efficiency unit of each strain was calculated as the diameter of the entire visible halo zone divided by the diameter of the zone with yeast colonies. To further investigate the influence of soluble phosphate on the phosphate-solubilizing abilities of yeasts, media containing three concentrations of exogenous soluble phosphate (0, 5, and 20 mM) were prepared using MS agar medium and 100 mM K_2_HPO_4_ solution. A yeast suspension (3 µL) was inoculated into (−P) MS agar plates containing each of the three soluble phosphate concentrations in triplicate. Before each experiment, yeasts were cultured on YPD agar plates (1% yeast extract, 2% peptone, 2% dextrose, and 2% (w/v) agar) at 28 °C in an incubator.

### Cocultivation of plants with yeasts in +P, –P or –P + calcium phosphate in vitro

Seeds of *Arabidopsis thaliana* ecotype Columbia (Col–0) and cherry type tomato were surface-sterilized using 1% (v/v) sodium hypochlorite solution with a few drops of Tween 20 for 5 min. After washing four times in sterile distilled water, seeds were sown in plates containing the Pi (+P), Pi-deficient (–P) or –P + calcium phosphate dibasic dehydrate (−P + Ca) medium. The + P, −P or −P + Ca contained quarter-strength MS medium (pH 5.7) and 0.5 g CPDD. The plates or vessels were then placed vertically in a plant growth chamber with a 16-hr light/8-hr dark photoperiod at 22 °C. Yeasts were inoculated in the agar containing 14-day-old germinated *Arabidopsis* or tomato seedlings (10 or four seedlings per plate or vessel, respectively).

### Cocultivation of plants with yeasts in +P, –P or –P + calcium phosphate in vivo

Seeds of cherry type tomato (*Solanum lycopersicum* var. cerasiforme) plants were sown in horticultural compost (Universal potting soil, FERTIPLUS, Helmond, The Netherlands). After germination, each plant was transplanted into a plastic pot containing soil. Plants were grown in a growth chamber under a 16-hr white light/8-hr dark cycle at 24 °C for 2 ~ 10 weeks. The light intensity was 180 µmoles photons m^−2^ s^−1^. Each potting plant was watered daily and supplemented with 10 mL P-derived solution. The plant was then treated with 5 mL of the yeast suspension (OD_660_ = 2) every 2 weeks.

### Isolation of total RNA

Total RNA was extracted from wild-type *Arabidopsis* with use of the RNeasy Plant Mini Kit (Qiagen, Hilden, Germany). RNA samples were treated with RQ1 RNase-Free DNase (Promega) to remove residual genomic DNA contamination, then purified and concentrated by use of the RNeasy MinElute Cleanup Kit (Qiagen). RNA samples were quantified with the MaestroNano (Maestrogen, Hsinchu, Taiwan). The integrity of RNA samples was confirmed by a 2% agarose gel electrophoresis displaying clear 28S and 18S bands. RNA with A260/280 and A260/230 ratios from 1.8 to 2.0 were used for analysis. RNA samples of three biological replicates were collected for the subsequent gene expression analysis.

### Real-time quantitative RT-PCR

Complementary DNA (cDNA) was synthesized from 2 µg total RNA by use of the iScript Reverse Transcription Supermix (Bio-Rad, Hercules, CA, USA) in a 20-µL reaction volume. The synthesized cDNA template was then diluted to two-fold, and identical amounts of cDNA input were used in PCR reactions. The fragments of target genes were amplified with gene-specific primers (Table 1). Each quantitative PCR reaction was analyzed in a 20-µL reaction volume containing an aliquot of 2 × Rotor-Gene SYBR Green Master Mix (Qiagen), 0.5 µM each forward and reverse primer, and 2 µL cDNA template. The *NbEF1* (*elongation factor 1*) gene was used as a reference. The PCR process was 95 °C for 2 min, 35 cycles at 95 °C for 15 s, 55 °C for 10 s, and 72 °C for 20 s (Rotor-Gene Q, Qiagen). The relative expression was calculated following the comparative 2^−ΔΔCT^ method (Yuan et al. 2006).

**Table 1:** List of primers and expected lengths of PCR products described in this study

Primers	Target gene	Forward primers (5′–3′)	Reverse primers (5′–3′)	Length (bps)	T_m_ (°C)
Phosphate starvation response (PSR) genes for real-time RT-PCR					
*AtPHT1;8*	*AT1G20860*	GTCTGAAGATGAGCCACAGATT	TCGTGTCTGAAGCAAAGTGCT	64	55
*AtG3PP1*	*AT3G47420*	TTGGGTCGACTATTGAGCAC	TGGACACGTTTTAGGAGCTTG	69	55
*AtIPK1*	*AT5G42810*	CGAGTCATGATTCTGCCCTTAT	ATCCGCATTTGGGCTTTATTTC	105	55
Reference genes for real-time RT-PCR					
*AtUBQ10*	*AT4G05320*	GGCCTTGTATAATCCCTGATGAATAAG	AAAGAGATAACAGGAACGGAAACATAGT	61	55

### Histochemical staining of GUS reporter activity

Transgenic *Arabidopsis* lines harboring the promoter-reporter gene fusions, including *proAtLBD29::GUS* (Okushima et al. 2007), *proAtPT2::GUS* (Alatorre-Cobos et al. 2014), and *proAtPLDZ2::GUS* (Cruz-Ramírez et al. 2006) constructs, were used to determine lateral root formation, phosphate deficiency, and phosphate level, respectively. The transgenic *Arabidopsis* lines were generated by *Agrobacterium*-mediated floral dip transformation (Zhang et al. 2006). GUS histochemical staining followed a protocol described earlier (Jefferson 1987). Samples of *Arabidopsis* roots or shoots were fixed with 4% paraformaldehyde in ice-cold 0.1 M phosphate buffer (pH 7.0) for 30 min, then incubated on ice and washed with phosphate buffer for 10 min. After washing three times, fixed tissues were placed in a desiccator and vacuum-infiltrated five times in histochemical GUS buffer containing 1 mM 5-bromo-4-chloro-3-indolyl β-D-glucuronide, 5 mM potassium ferricyanide, 5 mM potassium ferrocyanide, and 0.1% Triton X-100 in 100 mM sodium phosphate buffer (pH 7.0). Samples in buffer were incubated at 37 °C overnight in the dark. The chlorophyll was removed with 95% ethanol. GUS staining was examined under a stereomicroscope.

### Microscopy analysis

The shoots of transgenic *Arabidopsis* lines *(proPLDZ2::GUS* and *proPT2::GUS)* were rinsed briefly with water and then observed under a stereomicroscope (Z16 APO, Leica Microsystems, Heerbrugg, Germany). The lateral root formation of *Arabidopsis* AtLBD29 was examined under a light microscope (Leica DM 2500, Leica Microsystems, Wetzlar, Germany).

### Quantitative analysis of cellular pi in plants

Pi was measured at 14 days after inoculation and cellular Pi content was determined using the ascorbate method (AMSs, 1966). Briefly, plant tissues were weighed and subsequently submerged in 1 mL 1% glacial acetate. After eight freeze–thaw cycles, 100 mL of the extract was mixed with 200 mL H_2_O and 700 mL Pi reaction buffer (A = 0.48% NH_4_MoO_4_, 2.86% (v/v) H_2_SO_4_; B = 10% (w/v) ascorbic acid; A:B (v/v) = 6:1). The reaction was allowed to proceed at 45 °C for 20 min. Pi content was determined from the standard curve of K_2_HPO_4_ and is expressed as µM·g^− 1^ fresh weight.

### Statistical analysis

Data are expressed as mean ± standard deviation (SD). Differences between groups were assessed using Student *t* tests and analyses of variance. *P* < 0.05 was considered statistically significant.

## Results

### Phosphate-solubilizing ability

**Fig. 1:**
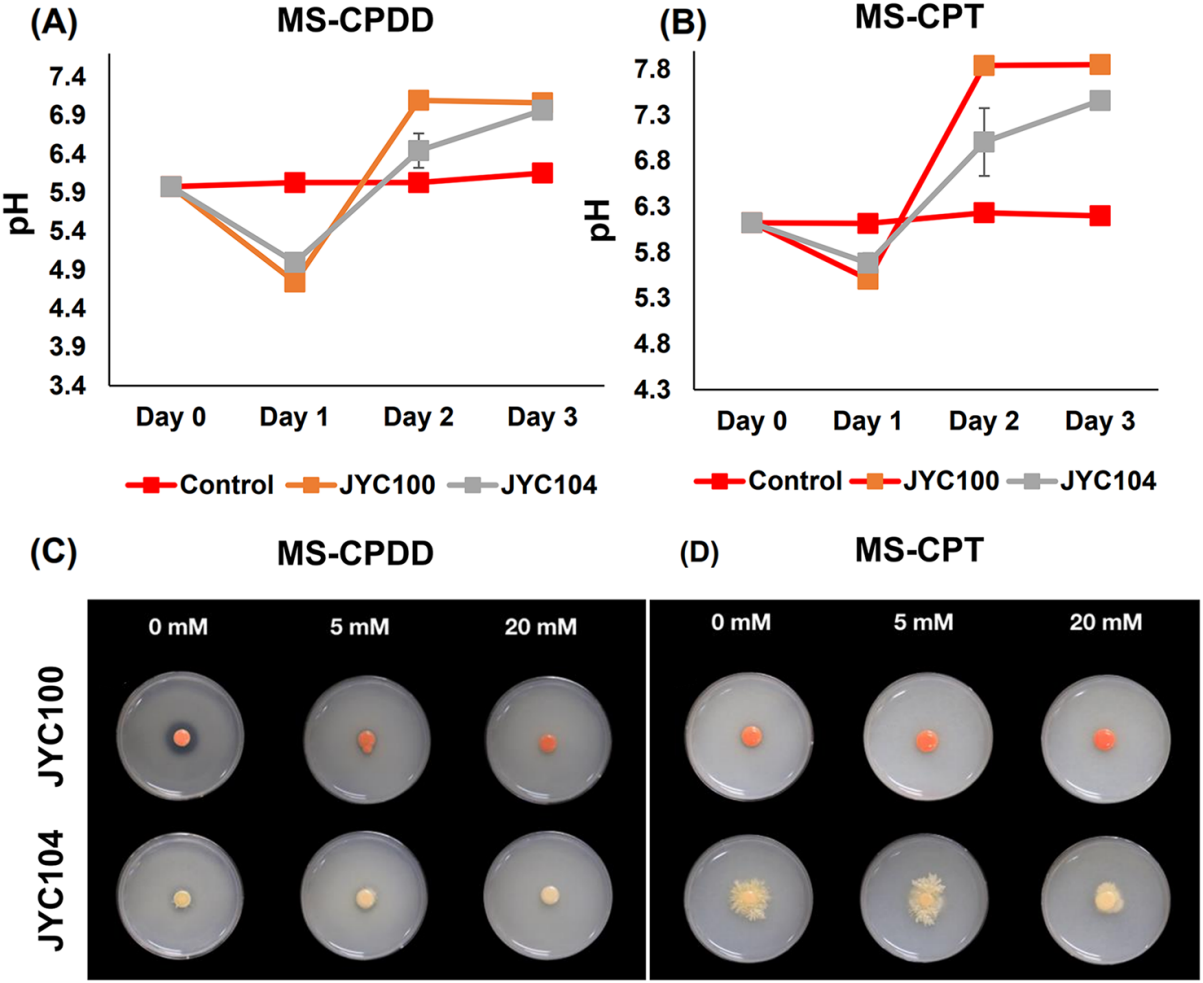
Changes in the pH and phosphate solubilizing activities of yeast cultures in Murashige–Skoog (MS) medium. **A** MS medium with calcium phosphate dibasic dihydrate (MS-CPDD). **B** MS medium with calcium phosphate tribasic (MS-CPT). **C** Colony and phosphate solubilization characteristics of yeast strains on MS plates with calcium phosphate dibasic dihydrate (MS-CPDD) and **D** calcium phosphate tribasic (MS-CPT). **C** and **D**, upper row: *Rhodosporidium paludigenum* JYC100; bottom row: *Aureobasidium pullulans* JYC 104

The yeast strain *R*. *paludigenum* JYC100 selected for this study exhibits dicalcium phosphate (DCP) and calcium phosphate tribasic (CPT) solubilizing activity in vitro on Pikovskaya’s agar plate, as reported in our previous study (Fu et al. 2016). *A*. *pullulans* JYC 104, in contrast, does not show solubilizing activity on either plate. In addition, in another earlier study, we found that the change in the pH of the medium is closely related to the phosphate-solubilizing ability of the yeasts (Kuo et al. 2018). More soluble phosphate was dissolved in the medium with low pH. In contrast, as shown in Fig. 1, these two yeast strains (JYC100 and JYC104, respectively) did not exhibit organic acid production that led to a decrease in the pH of the medium (Fig. 1A and B, orange and grey lines, respectively). To further confirm the phosphate-solubilizing ability of these two yeasts, we inoculated them on (−P) MS-CPDD and MS-CPT plates in this study. We found that *R*. *paludigenum* JYC100 exhibited a clear zone on MS-CPDD and MS-CPT plates with null levels of exogenous soluble phosphate (0 mM). *A*. *pullulans* JYC 104 also showed weak solubilizing activity on MS-CPDD but not on MS-CPT plates with null of exogenous soluble phosphate (0 mM). When the soluble phosphate concentration increased, the diameters of the clear zones decreased (Fig. 1C, D). This suggests that the yeast’s calcium phosphate-solubilizing ability was affected by exposure to high concentrations of soluble phosphate. In summary, these findings indicate that the principal mechanisms underlying Pi solubilization by these two yeasts is not caused by organic acid secretion.

### Effects of yeasts on* A. thaliana* growth in +P, –P or –P + calcium phosphate plates

**Fig. 2:**
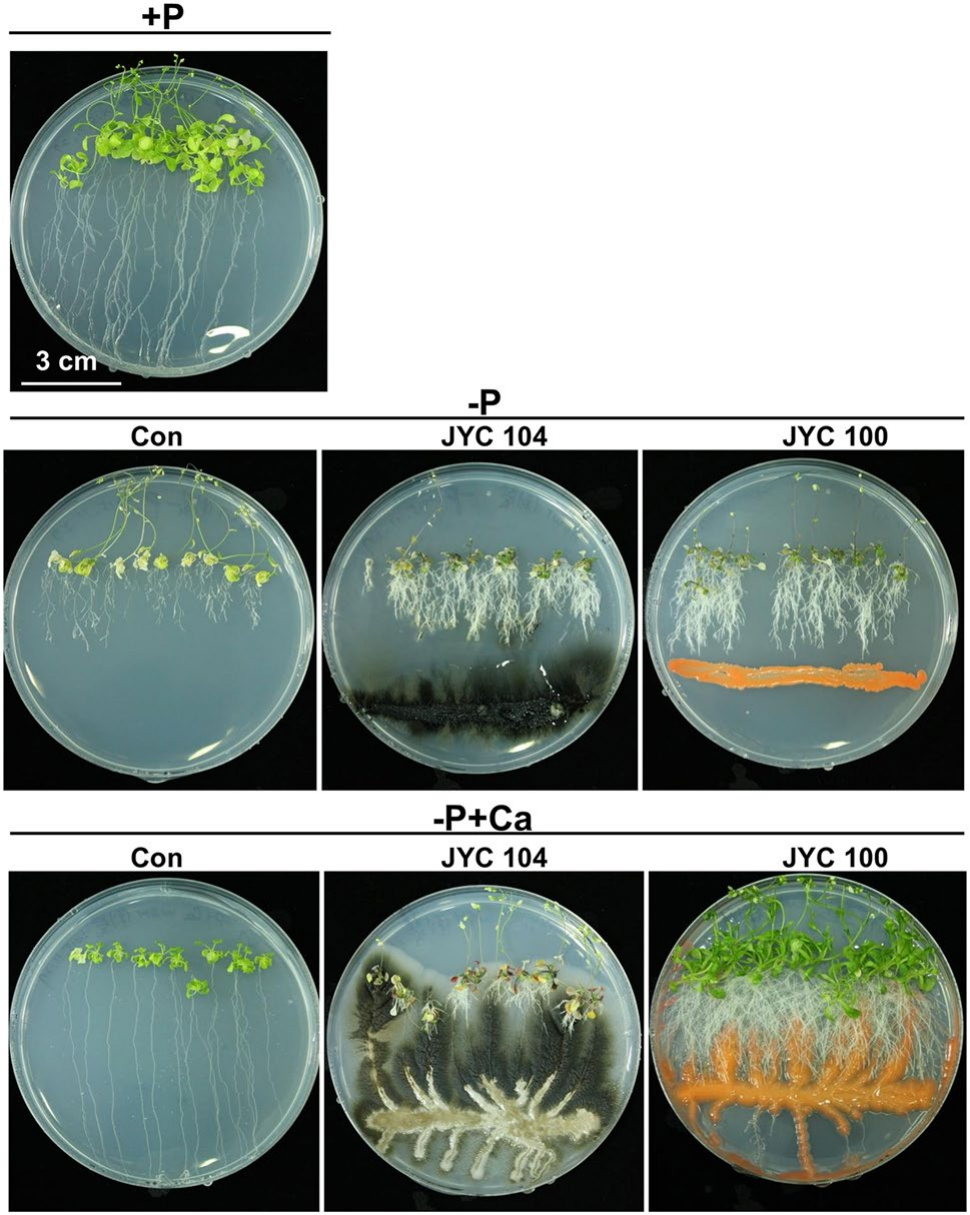
Effects of phosphate-solubilizing yeasts on* A. thaliana* growth on –P + calcium phosphate dibasic dihydrate (−P + Ca) plates. After 2 weeks of cocultivation of *A. thaliana*, remarkable increases in biomass were observed in plants with *Rhodosporidium paludigenum* JYC100 (right panel) compared to those with *Aureobasidium pullulans* JYC 104 (middle panel) and controls (left panel). It indicated that plants were inoculated with *R*. *paludigenum* JYC100 exhibiting high phosphate-solubilizing ability and *A*. *pullulans* JYC 104 exhibiting weak phosphate-solubilizing ability. +P, containing Pi; –P, Pi-deficient

**Fig. 3:**
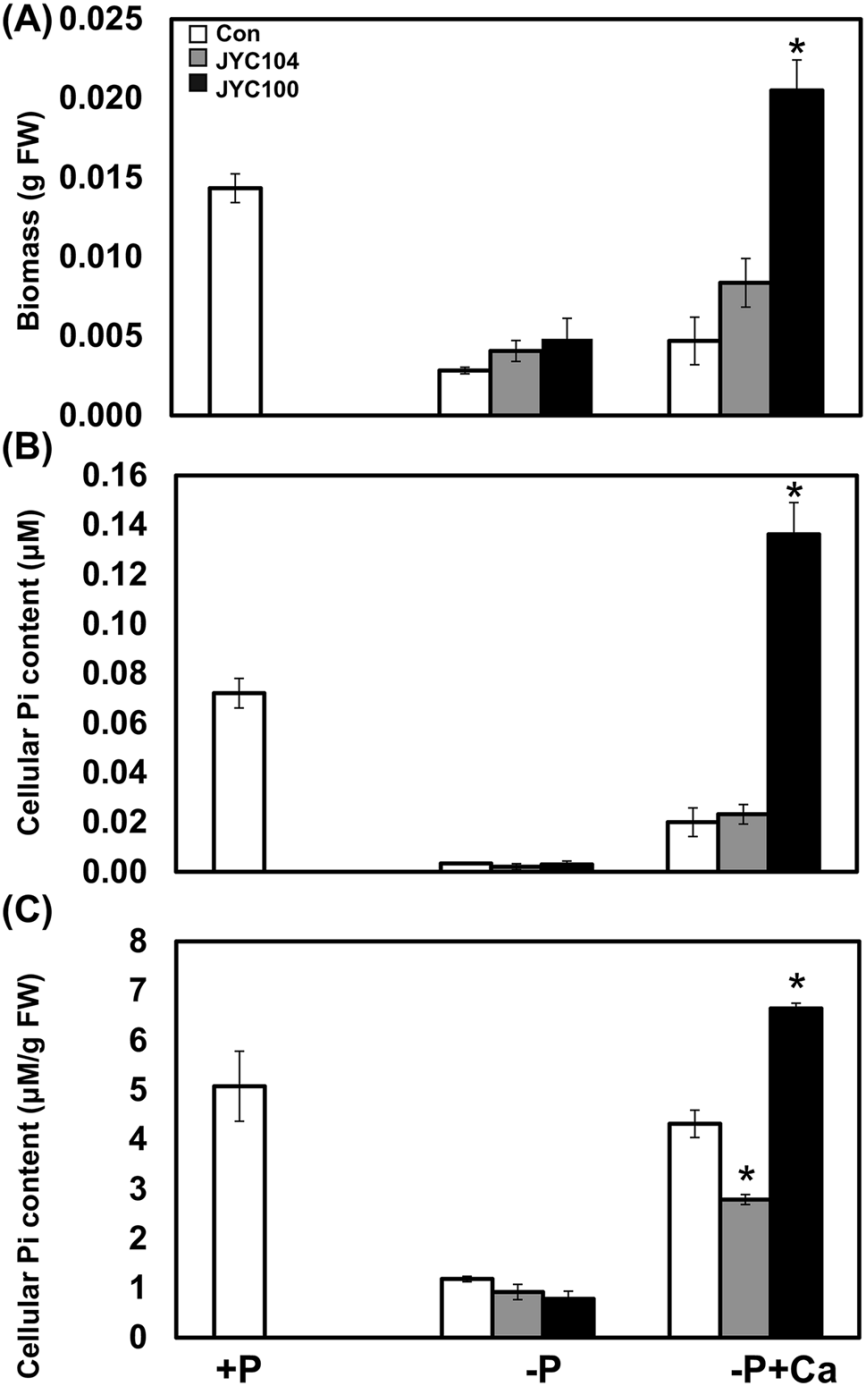
Biomass and cellular Pi content in *A. thaliana* on–P + calcium phosphate dibasic dihydrate (−P + Ca) plates. **A** Biomass. **B** cellular Pi content in µM. **C** cellular Pi content in µM/g fresh weight of *A. thaliana* in control (white bars) and yeast-treated groups (grey bars: *Aureobasidium pullulans* JYC 104; black bars: *Rhodosporidium paludigenum* JYC100). Data are the mean of three independent experiments ± SD. Means in the same group with the same letter are not significantly different from each other (Student *t* tests). Con, control

To understand the effects of phosphate-solubilizing yeasts on plant growth and development, we used *A*. *thaliana* as a model system. Wild-type *A*. *thaliana* seedlings were germinated and grown on MS-CPDD plates. At 2 weeks after germination, yeast strains were inoculated 3 cm from the root tips; one plate was not inoculated to serve as a negative control. After 2 weeks of cocultivation of *A*. *thaliana*, remarkable increases in biomass were observed in plants with *R*. *paludigenum* JYC100 (Fig. 2, right panel, label −P + Ca in bottom row; Fig. 3 A, black bar, label −P + Ca) compared to those with *A*. *pullulans* JYC 104 (Fig. 2, middle panel, label −P + Ca in bottom row; Fig. 3 A, grey bar, label −P + Ca) and controls (Fig. 2, left panel, label −P + Ca in bottom row; Fig. 3 A, white bar, label −P + Ca). In particular, the cellular P_i_ content of *A*. *thaliana* cocultivated with *R*. *paludigenum* JYC100 (Fig. 3B and C, black bars, label −P + Ca) was much higher than that of *A*. *thaliana* cocultivated with *A*. *pullulans* JYC 104 (Fig. 3B and C, grey bars, label −P + Ca) or the control group (Fig. 3B and C, white bars, label −P + Ca). These results suggest that *R*. *paludigenum* JYC100, with its phosphate-solubilizing ability, exerts beneficial effects on plant growth and development. However, *A*. *pullulans* JYC 104, which also showed weak phosphate-solubilizing abilities, did not show plant development-promoting behavior in our plant–yeast cocultivation assay.

### Phosphate-solubilizing yeasts regulate root system architecture in *Arabidopsis*

**Fig. 4:**
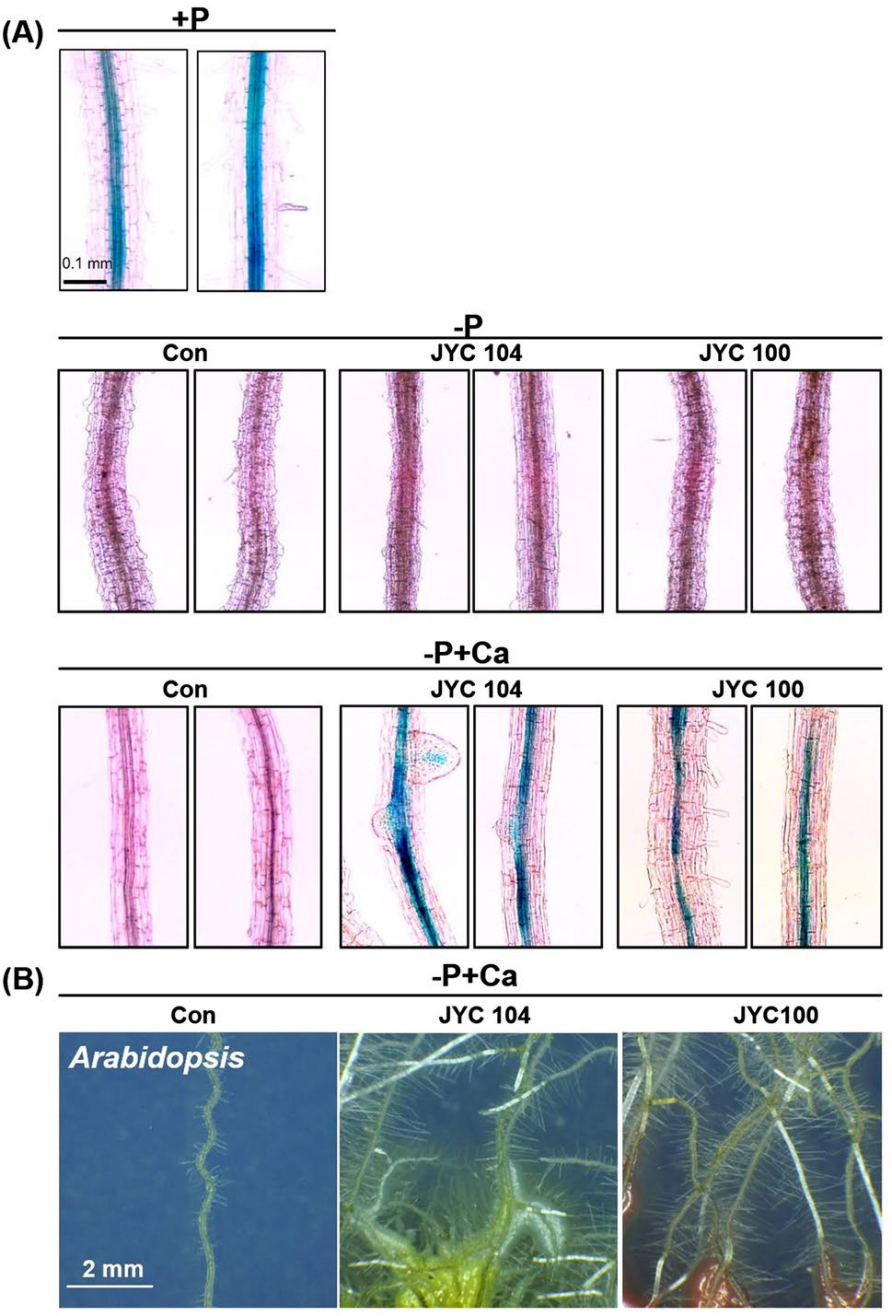
Histochemical analysis of GUS activity driven by the lateral organ boundariesdomain-29 promoter in the *Arabidopsis proAtLBD29::GUS *line. **A** *Arabidopsis* seeds were sown on + P (upper row), −P (middle row) and –P + Ca (bottom row) medium and grown for 2 weeks. The seedlings were then inoculated with the yeasts JYC104 (middle panel) and JYC100 (right panel) and cocultivated for another 2 weeks. *proAtLBD29::GUS* expression was assessed with a light microscope. Scale bar, 0.1 mm. **B** Lateral root formation observed after cocultivation with the yeasts JYC104 (middle panel) and JYC100 (right panel) for 2 weeks in –P + Ca medium. The photographs of lateral roots and root hairs were taken with a stereomicroscope. Scale bar, 2 mm. +P, containing Pi; –P, Pi-deficient; −P + Ca, –P + calcium phosphate dibasic dihydrate; Con, control

To obtain further insights into the effects of phosphate-solubilizing yeasts on root system architecture, the steles of primary roots, the development of lateral roots, and the formation of root hairs of *Arabidopsis* seedlings cocultivated with the yeasts were assessed (Fig. 4). Transgenic *proAtLBD29::GUS Arabidopsis* seedlings were cocultivated with the various yeast strains. *AtLBD29* is an auxin-responsive gene and expressed predominately in root stele, and the *proAtLBD29::GUS* line has been used to monitor the development of lateral roots (Okushima et al. 2007). Histochemical staining of the primary roots was performed on transgenic *proAtLBD29::GUS* seedlings (Fig. 4A). The region of the root stele that was GUS-stained was obvious in transgenic *proAtLBD29::GUS* plants supplemented with P (+P) but not in those under P starvation (–P) conditions. However, GUS was significantly expressed in the steles of *proAtLBD29::GUS* plants in MS-CPDD medium (–P + Ca) cocultivated with JYC104 (Fig. 4A, middle panel in bottom row) and JYC100 (Fig. 4A, right panel in bottom row) compared with those of the control plants (Fig. 4A, left panel in bottom row). The development of lateral roots and root hairs was significantly stimulated by cocultivation with JYC104 (Fig. 4B, middle panel) and JYC100 (Fig. 4B, right panel) compared with that seen in the controls (Fig. 4B, left panel). An increased GUS staining was observed when the yeasts JYC104 and JYC100 were present in plants growing in −P + Ca medium suggesting that under those conditions, a higher expression of GUS promoted by *proAtLBD29*, occurred.

### Analysis of promoter activity of the phosphate transporter *AtPT2* in *Arabidopsis* plants cocultivated with phosphate-solubilizing yeast

**Fig. 5:**
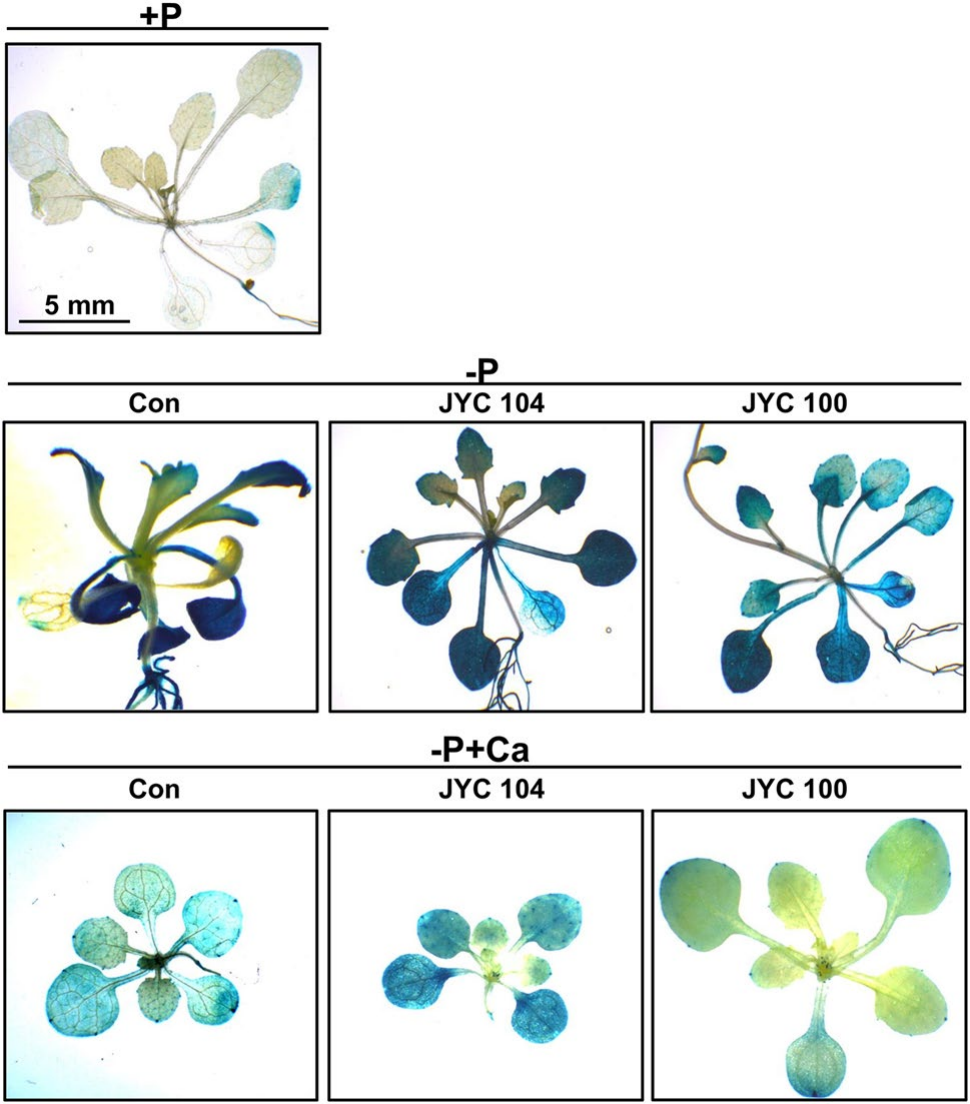
Histochemical analysis of GUS activity driven by the *AtPT2* (Phosphate Transporter 2) promoter from an *AtPT2::GUS* construct under different P conditions. *Arabidopsis proAtPT2::GUS* seedlings were grown under –P, +P or –P + Ca conditions for 2 weeks. The plants were inoculated with the yeasts JYC104 (middle panel) or JYC100 (right panel) at the bottom of the medium plates. After cocultivation of the plants with yeasts for 2 weeks, GUS activity in leaves was monitored by histochemical analyses. The GUS activity of the plants grown in –P + Ca medium was lower when cocultivated with the yeast JYC100 (right panel) than when cocultivated with JYC104 (middle panel) and lower than that of controls (left panel) Scale bar, 5 mm. +P, containing Pi; –P, Pi-deficient; −P + Ca, –P + calcium phosphate dibasic dihydrate; Con, control

To analyze the promoter activity of *AtPT2* in response to phosphate, a GUS assay was performed for the transgenic *proAtPT2::GUS* of *Arabidopsis* grown in a medium containing various levels of phosphate (–P, +P or –P + Ca). It has been previously documented that the *proAtPT2::GUS* expression was induced by P deficiency and served as a molecular marker for P status (Alatorre-Cobos et al. 2014). We found that GUS activity in shoot tissue was increased in response to P deficiency (–P) (Fig. 5, middle row), compared to the plants supplemented with P (+P) (Fig. 5, upper row). Noticeably, the GUS activity of the plants grown in –P + Ca medium was lower when cocultivated with the yeast JYC100 (Fig. 5, right panel in bottom row) than when cocultivated with JYC104 (Fig. 5, middle panel in bottom row) and lower than that of controls (Fig. 5, left panel in bottom row). This suggests that the *Arabidopsis* plants were able to acquire sufficient P in –P + Ca medium cocultivated with the yeast JYC100. Yeast JYC100 may thus be beneficial for plant growth, by releasing P from the –P + Ca medium.

### Analysis of phosphate acquisition efficiency of *Arabidopsis* plants cocultivated with phosphate-solubilizing yeasts

**Fig. 6:**
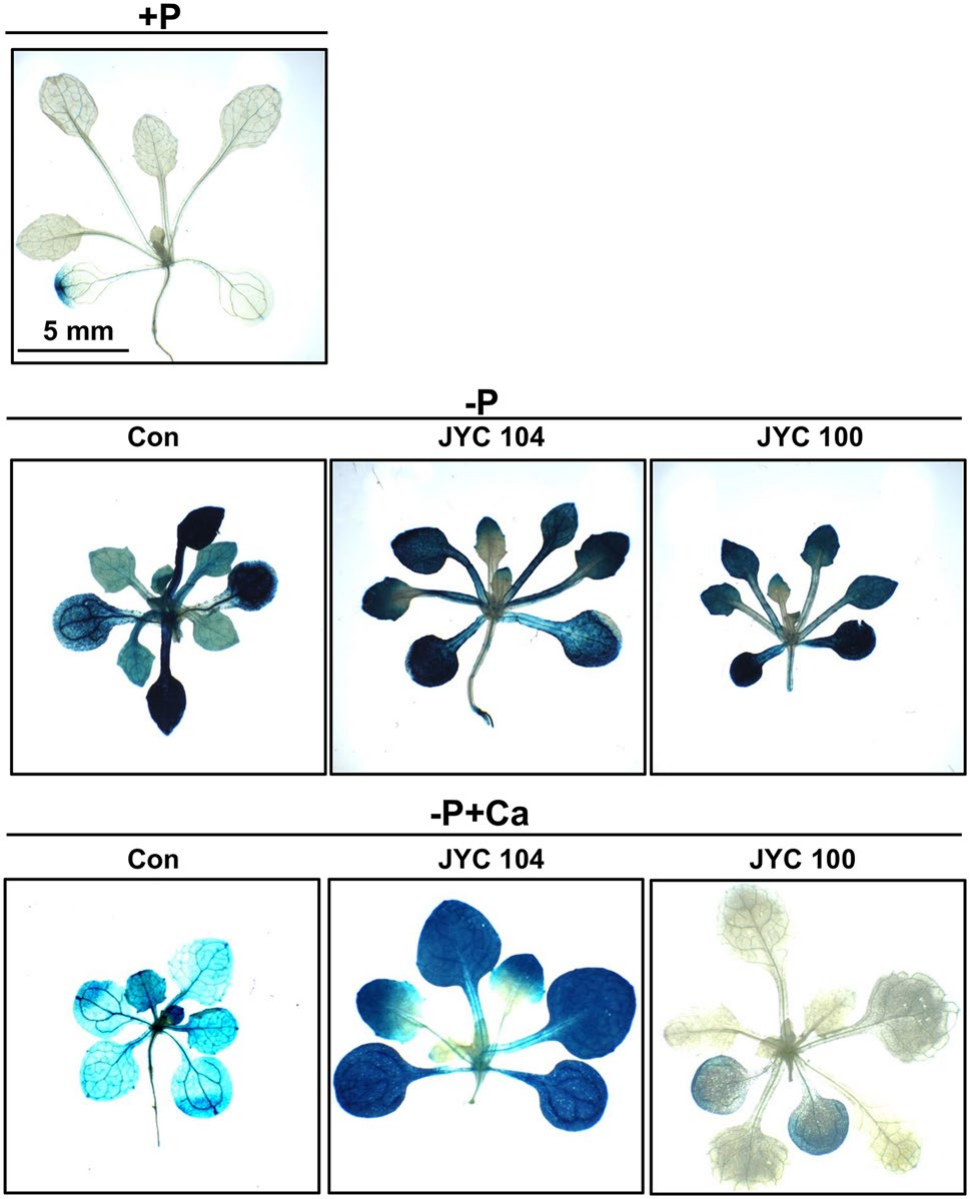
Analysis of *AtPLDZ2* (Phospholipase DZ2) promoter activity in *Arabidopsis* plants under different P conditions. A histochemical GUS analysis of seedlings grown under adequate P supply (+P) showed only a trace of GUS staining in the margin of the old leaves (upper row). When *Arabidopsis* plants were cocultivated with the yeasts grown under P deficiency (–P) (middle row), a dramatic increase in GUS staining was detected, compared to the staining observed in plants in + P medium. In –P + Ca medium (bottom row), GUS staining was clearly detected in the control (left panel) and the plants cocultivated with the yeast JYC104 (middle panel). In contrast, there was very weak GUS staining in leaf tissues of *Arabidopsis* plants cocultivaed with the yeast JYC100 (right panel) (Fig. 6). These findings suggest that the *Arabidopsis* plants acquired sufficient P from –P + Ca medium when cocultivated with the yeast JYC100. +P, containing Pi; –P, Pi-deficient; −P + Ca, –P + calcium phosphate dibasic dihydrate; Con, control

To investigate the phosphate acquisition efficiency of *Arabidopsis* in more detail, a *proPLDZ2::GUS* transgenic line with a transcriptional gene fusion between the *AtPLDZ2* promoter and *GUS* (Cruz-Ramírez et al. 2006) was used. The promoter activity of *AtPLDZ* is increased in both shoots and roots during P starvation (Cruz-Ramírez et al. 2006). A histochemical GUS analysis of seedlings grown under adequate P supply (+P) showed only a trace of GUS staining in the margin of the old leaves (Fig. 6, upper row). When *Arabidopsis* plants were cocultivated with the yeasts grown under P deficiency (–P) (Fig. 6, middle row), a dramatic increase in GUS staining was detected, compared to the staining observed in plants in + P medium. In –P + Ca medium (Fig. 6), GUS staining was clearly detected in the control (Fig. 6, left panel in bottom row) and the plants cocultivated with the yeast JYC104 (Fig. 6, middle panel in bottom row). In contrast, there was very weak GUS staining in leaf tissues of *Arabidopsis* plants cocultivaed with the yeast JYC100 (Fig. 6, right panel in bottom row). Taken together, these findings suggest that the *Arabidopsis* plants acquired sufficient P from –P + Ca medium when cocultivated with the yeast JYC100.

### Analysis of the mRNA levels of P starvation-responsive genes in *Arabidopsis* plants cocultivated with phosphate-solubilizing yeasts

**Fig. 7:**
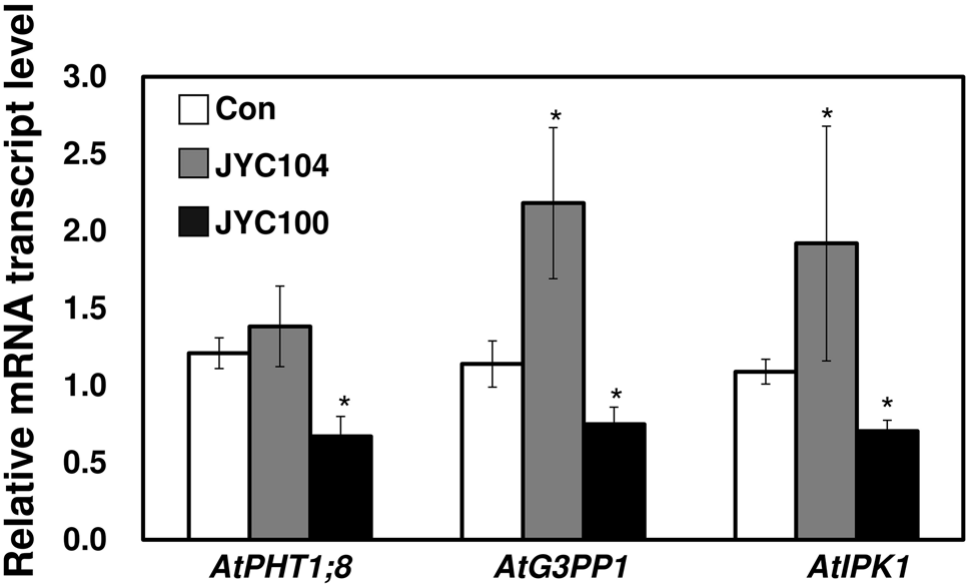
Relative mRNA transcript level of marker genes for P starvation in *Arabidopsis* plants grown under different P conditions. Real-time quantitative RT-PCR of mRNA level of P starvation-induced genes, including *AtPHT1;8*, *AtG3PP1*, and *AtIPK1*, in *Arabidopsis* plants. Total RNA was isolated from leaves of 4-week-old plants in + P, −P and –P + Ca medium cocultivated with the yeasts JYC104 (grey bars) or JYC100 (black bars). The cDNA was synthesized by reverse transcription of total RNA. *AtUBQ10* was used as an internal amplification control for qRT-PCR. *P < 0.05 compared with the control determined by fold changes and Student *t* tests (P < 0.05). The relative mRNA level was compared to that of control plants (white bars) and presented in arbitrary units. The analyses are based on three biological replicates. +P, containing Pi; –P, Pi-deficient; −P + Ca, –P + calcium phosphate dibasic dihydrate; Con, control

Transcriptional analysis provides new insights into plants responses to phosphate starvation. *Arabidopsis* plants were cocultivated with the phosphate-solubilizing yeasts. The mRNA levels of P starvation-induced genes in the plants were examined with real-time quantitative reverse transcription-PCR (qRT-PCR). Analyses of the mRNA profiles of three genes involved in the physiological (*AtPHT1;8*) (Mudge et al. 2002), metabolic (*AtG3PP1*) (Ramaiah et al. 2011), and transcriptional (*AtIPK1*) (Kuo et al. 2014) responses to P starvation were carried out on the plants that were grown in –P + Ca medium. Compared to the control (Fig. 7, white bars), the mRNA levels of the three genes was induced by the yeast JYC104 (Fig. 7, grey bars). In contrast, a significant decrease in the transcription levels of the three genes was observed when the plants were cocultivated with JYC100 (Fig. 7, black bars). This finding indicates that *Arabidopsis* plants cocultivated with the yeast JYC104 suffer from P starvation. The yeast JYC100 thus contributes to the maintenance of P homeostasis in *Arabidopsis*.

### In vitro and in planta plant growth promotion assay of tomato plants cocultivated with phosphate-solubilizing yeasts

**Fig. 8:**
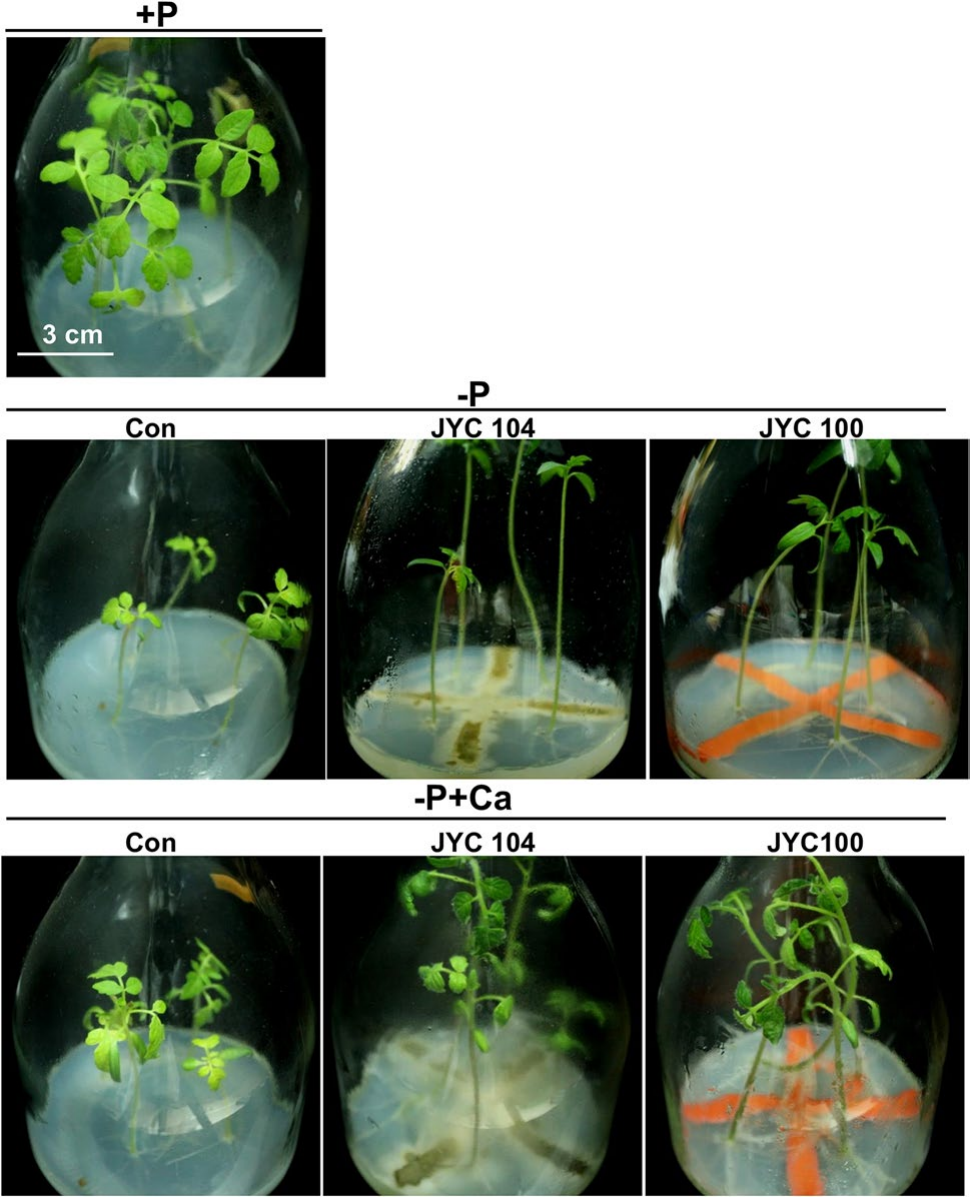
Effects of phosphate-solubilizing yeasts on the tomato plants grown in vitro under + P (upper row), −P (middle row) and –P + Ca (bottom row) conditions. Morphology of 20-day-old tomato plant after cocultivation with the yeasts JYC104 (middle panel) or JYC100 (right panel) for 10 days. Small and stunted growth was observed under the –P conditions (middle row) as compared to plants grown in the + P medium (upper row). However, the growth promotion of the tomato plants was more pronounced in the presence of JYC100 (right panel) in –P + Ca medium (bottom row) as compared to the presence of JYC104 (middle panel) and control (left panel) plants. +P, containing Pi; –P, Pi-deficient; −P + Ca, –P + calcium phosphate dibasic dihydrate; Con, control

**Fig. 9:**
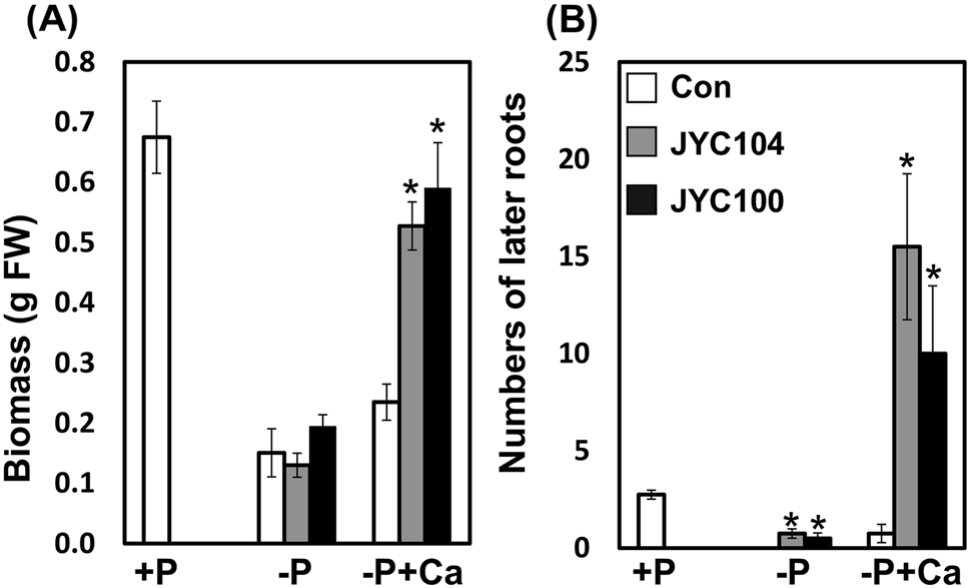
Effects of the yeast strains JYC104 or JYC100 on the tomato plants grown in vitro. **A** Biomass and **B** number of lateral roots of the tomato plants after 10 days of interactions with the yeasts. The biomass of the plants cocultivated with the yeasts JYC104 (grey bars) and JYC100 (black bars) was higher than that of the controls (white bars) in –P + Ca medium. Lateral root formation was also increased when plants were cocultivated with both of the yeast strains compared to the control plants in –P + Ca medium. The data are mean ± SE. *P < 0.05 within each P condition. Three biological replicates were performed with similar results. Con, control

**Fig. 10:**
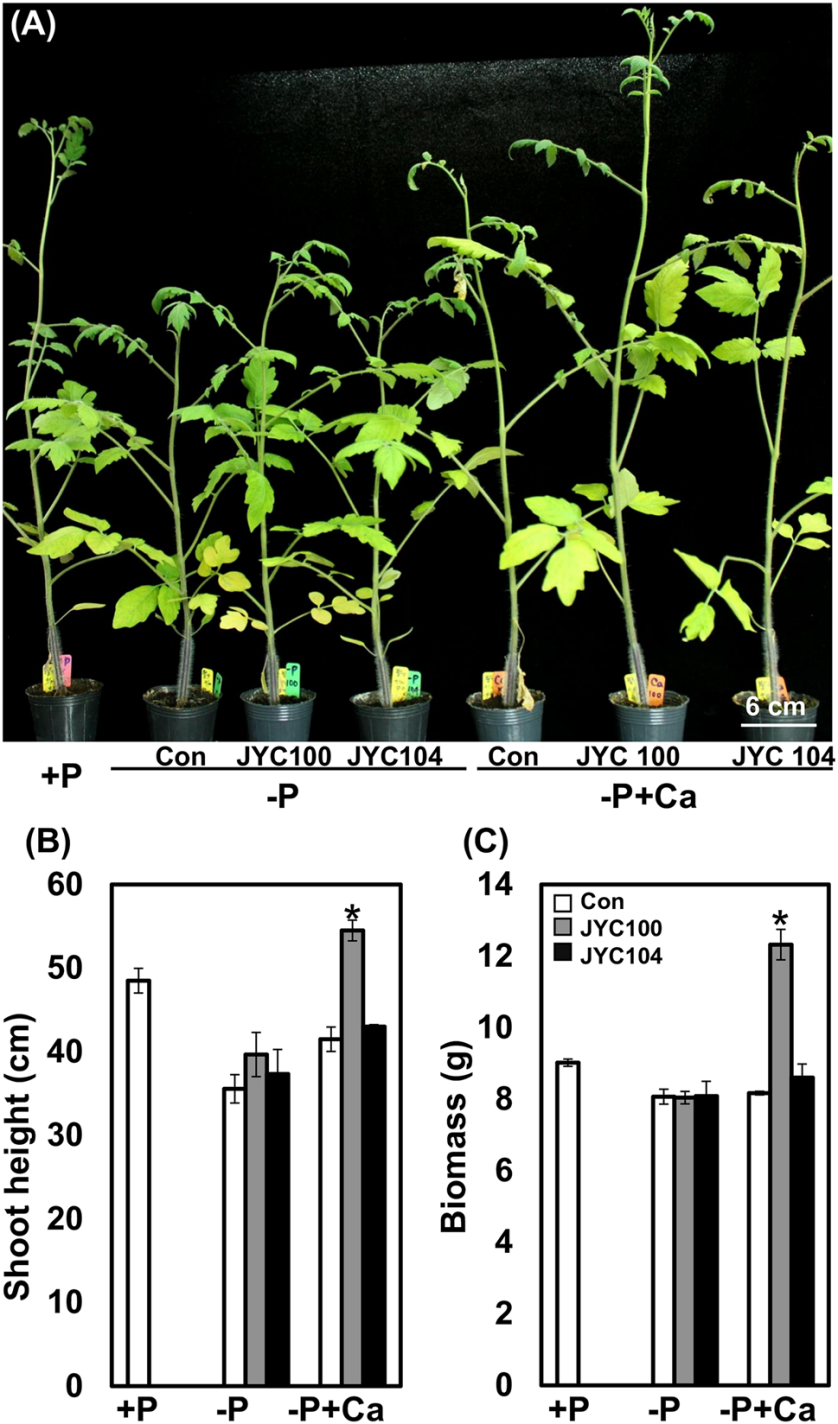
Influence of phosphate-solubilizing yeasts on the tomato plants grown in potting soil under + P, −P and –P + Ca conditions. **A** Morphology of 10-week-old tomato plant after cocultivation with the yeasts JYC104 or JYC100 for 8 weeks. The individual plant in the pot was treated with 5 mL of the yeast suspension (OD_660_ = 2) every 2 weeks. **B** Shoot height and **C** biomass of the tomato plants after 10 weeks of interactions with the yeasts. The plant growth promotion was observed when the tomato plants were grown in the potting soil under P deficiency (–P) supplemented with Ca and inoculated with the yeast JYC100. The −P + Ca plants treated with the yeast JYC100 (grey bars) also exhibited a significant increase in shoot height (**B**) and biomass (**C**), compared to plants cocultivated with the yeast JYC104 (black bars) and control (white bars) plants. The data are mean ± SE. *P < 0.05 within each P condition as compared to the control. Three biological replicates were performed with similar results. +P, containing Pi; –P, Pi-deficient; −P + Ca, –P + calcium phosphate dibasic dihydrate; Con, control

We extended the study to evaluate the beneficial effects of the phosphate-solubilizing yeasts on tomato plants. Tomato plants were grown in MS medium supplemented with either P (+P), −P, or –P + Ca. The tomato plants were then inoculated with the yeasts JYC104 and JYC100 in vitro. Small and stunted growth was observed under the –P conditions (Fig. 8, middle row) as compared to plants grown in the + P medium (Fig. 8, upper row). However, the growth promotion of the tomato plants was more pronounced in the presence of JYC100 in –P + Ca medium (Fig. 8, right panel in the bottom row) as compared to the presence of JYC104 (Fig. 8, middle panel in the bottom row) and control (Fig. 8, left panel in the bottom row) plants. The biomass of the plants cocultivated with the yeasts JYC104 (Fig. 9A, grey bar) and JYC100 (Fig. 9A, black bar) was higher than that of the controls (Fig. 9A, white bar) in –P + Ca medium. Lateral root formation was also increased when plants were cocultivated with both of the yeast strains (Fig. 9B, black and grey bars) compared to the control plants (Fig. 9B, white bar) in –P + Ca medium. Furthermore, plant growth promotion was also observed when the tomato plants were grown in the potting soil under P deficiency (–P) supplemented with Ca and inoculated with the yeast JYC100 (Fig. 10 A). The −P + Ca plants treated with the yeast JYC100 (grey bars) also exhibited a significant increase in shoot height (Fig. 10B) and biomass (Fig. 10 C), compared to plants cocultivated with the yeast JYC104 (black bars) and control (white bars) plants. Taken together, these findings suggest that the yeast JYC100 could help maintain adequate supplies of P, thereby leading to growth promotion of tomato plants in –P + Ca medium both in vitro and in vivo.

## Discussion

In this study on the abilities of different yeasts to promote the growth of plants, we found that the diameters of the visible halo zones around the colonies disappeared when yeasts were incubated with the soluble phosphate (5 and 20 mM). This suggests that the phosphate-solubilizing ability of yeasts is affected by exposure to soluble phosphate. In contrast, in a previous study, we found that some yeast strains still dissolve calcium phosphate in the presence of a high concentration of soluble phosphate (Kuo et al. 2018). In 2017, Zeng et al. reported that the growth and mineral phosphate solubilization of a phosphate-solubilizing bacteria (PSB), *Burkholderia multivorans* WS-FJ9, can be affected by exogenous soluble phosphate (Zeng et al. 2017). They found that the genes involved in glucose metabolism and sugar ABC-type transporter were continuously down-regulated. This finding demonstrates that the metabolic channeling of glucose towards the phosphorylative pathway is negatively regulated by soluble phosphate. In 2020, the same authors further investigated eight P-related genes under different exogenous soluble phosphate conditions (Liu et al. 2020), and found that these genes are not only related to and independent of each other in the solubilization of organic phosphate and Pi but also support each other’s roles. Similarly, Yang et al. also found that the phosphate solubilization genes (glucose dehydrogenase; GDH) of PSB *Pseudomonas* sp. Wj1 and *Enterobacter* sp. Wj3 have different expression patterns at different soluble phosphate levels (Yang et al. 2016). This suggests that these PSMs have evolved mechanisms to sense the concentration of exogenous soluble phosphate and to adapt to different phosphate source environments. They can regulate their phosphate-solubilizing ability through fine-tuning metabolic pathways, but the response is strain-dependent. Thus, those strains who do have regulation abilities are great candidates for biofertilizer, because they do not invest energy in producing organic acids or related enzymes and can instead use the available energy to increase their fitness.

Here we found that *R*. *paludigenum* JYC100 with phosphate-solubilizing abilities exerts beneficial effects on plant growth and development. However, *A*. *pullulans* JYC 104, which also showed weak phosphate-solubilizing abilities, did not show this plant growth promoting trait. We suggest that the IAA produced by *R*. *paludigenum* JYC100 is not the main reason for the observed effects on plant growth and development, since the IAA-producing abilities of *A*. *pullulans* JYC 104 are even better than those of *R*. *paludigenum* JYC100. In our previous study, *R*. *paludigenum* JYC100 produced 400.59 ± 52.5 µg/mL IAA in the presence of tryptophan (Trp), a prominent precursor of IAA. *A*. *pullulans* JYC 104 can produce 610.63 ± 54.7 µg/mL IAA in medium with Trp. Both strains are still able to produce IAA in the absence of exogenous Trp (39.62 ± 3.2 and 145.63 ± 12.9 µg/mL). However, we cannot completely rule out IAA as the reason for the effect, since the medium for IAA production assays (YPD medium) is different from that used in the yeast–plant cocultivation experiment (MS medium).

Besides solubilizing P, some PSMs also demonstrate potential as biocontrol agents against some plant pathogens. The present study indicated that *R*. *paludigenum* JYC100 can regulate phosphate solubilization according to exogenous soluble phosphate. Second, *R*. *paludigenum* JYC100 significantly enhanced the growth of specific plants. Previous studies also indicated that *R*. *paludigenum* can significantly inhibit various fungal pathogens of harvested fruits (Wang et al. 2010a; Wang et al. 2010b; Lu et al. 2013). In addition, *R*. *paludigenum* is one of the most interesting IAA-producers (Nutaratat et al. 2015). As mentioned above, *R*. *paludigenum* JYC100 produces high amounts of IAA in the presence of Trp, and is still able to produce IAA in the absence of exogenous Trp (Fu et al. 2016). Last but not least, *R*. *paludigenum* is a kind of single-celled aquatic organism, which is widely distributed in the sea and various rivers, also called “marine red yeast”. This species can sometimes arise from any plant-associated environment (Fu et al. 2016; Nutaratat et al. 2015). It has been shown that marine microorganisms have superior characteristics compared to their terrestrial counterparts, with especially marine yeasts showing higher tolerance to salts and many inhibitors usually present in fermentation media (Zaky et al. 2018; Greetham et al. 2019). *R*. *paludigenum* has even been demonstrated to have excellent fermentation capabilities compared with the reference terrestrial yeast and other different yeasts species (Nutaratat et al. 2015; Nutaratat et al. 2016; Liu et al. 2021). These results demonstrate the potential of *R*. *paludigenum* for biofertilizer applications and for use using in sustainable agricultures. However, field trials should be conducted, and the mechanisms underlying the calcium-phosphate-solubilizing activity of *R*. *paludigenum* JYC100 require further study to verify the practical and effective use of this yeast train as a biofertilizer.

Lateral roots are important for uptake of nutrients due to their ability to increase the area of a root system (López-Bucio et al. 2003). Lateral roots originate from a subset of pericycle cells localized at the outer ring of the stele (Péret et al. 2009). In this study, both of the phosphate-solubilizing yeasts JYC104 and JYC100 were able to promote lateral root formation in plants (Figs. 4 and 8). In *Arabidopsis*, lateral root density and length were increased when the plants were cocultivated with the two yeast strains. This was demonstrated by an increase in GUS activity of *proAtLBD29::GUS* in the root stele (Fig. 4). It has been found that LBD29 regulates the cell cycle progression during lateral root formation (Feng et al. 2012). Thus, the two yeast strains might stimulate the cell cycle in the root stele and subsequently led to lateral root formation. Induction of lateral root formation was also observed in tomato plants cocultivated with yeasts JYC104 or JYC100 (Fig. 8). However, only the yeast JYC100 was able to maintain P homeostasis and promote plant growth in –P + Ca conditions in *Arabidopsis* (Fig. 2) and tomato (Fig. 8). These findings indicate the dual roles of yeast JYC100, one related to their phosphate-solubilizing capacity and one to the promotion of lateral roots. The yeast JYC100 thus also possesses the potential to help with P acquisition for economically important vegetables and fruits.

Many studies have been conducted on the phosphate-solubilizing abilities of microorganisms (Soumare et al. 2020). However, there is a lack of evidence of a correlation between growth-promotion of plants and the yeast-mediated phosphate solubilization under coculture conditions. In this study, the P homeostasis of *Arabidopsis* plants cocultivated with the phosphate-solubilizing yeasts was examined in terms of morphology, physiology, metabolism, and transcriptional regulation. Morphological analysis of the *Arabidopsis* plants showed an increase in biomass (Fig. 2) and cellular Pi content (Fig. 3) when cocultivated with JYC100 under –P + Ca conditions. The results were further validated by the promoter activity of phosphate transporter *AtPT2* (Fig. 5) and mRNA level of *AtPHT1;8* (Fig. 7) in adequate P supply by the yeast JYC100. The mRNA levels of metabolism-related genes such as *AtPLDZ2* (Fig. 6) and *AtG3PP1* (Fig. 7) demonstrated that sufficient P was supplied in the *Arabidopsis* plants treated with the yeast JYC100. The *AtIPK1* gene modulates phosphate homeostasis at the transcriptional level (Kuo et al. 2014). The steady-state mRNA level of *AtIPK1* was unchanged in the *Arabidopsis* plants (–P + Ca) inoculated with yeast the JYC100 (Fig. 7). Although the yeast JYC104 exhibited minimum phosphate-solubilizing capacities, it inhibited plant growth (Fig. 2) and led to P starvation (Figs. 5, 6 and 7). This suggests that only some of the phosphate-solubilizing yeasts are able to enhance plant growth under coculture conditions. Several lines of evidence indicate that the unique phosphate-solubilizing yeast JYC100 cannot only help *Arabidopsis* plants increase their biomass but also maintain cellular P homeostasis.

## Conclusion

In the present study, the phosphate-solubilizing red yeast *R*. *paludigenum*, with fine-tuning capabilities was isolated and used as inoculants for plant growth promotion in *Arabidopsis* and tomato plants. Results indicated significant levels of growth enhancement by the isolate in seedling assay and greenhouse experiment. The positive influence can be owed to solubilization of fixed inorganic phosphates in soil as well as release of plant growth hormones. Thus, *R*. *paludigenum* JYC100 represents a promising candidate for biofertilizer development in regions with high calcium-bound phosphate levels.
